# The NEET and Hikikomori spectrum: Assessing the risks and consequences of becoming culturally marginalized

**DOI:** 10.3389/fpsyg.2015.01117

**Published:** 2015-08-18

**Authors:** Yukiko Uchida, Vinai Norasakkunkit

**Affiliations:** ^1^Kokoro Research Center, Kyoto UniversityKyoto, Japan; ^2^Department of Psychology, Gonzaga UniversitySpokane, WA, USA

**Keywords:** NEET, Hikikomori, marginalization, culture, risk factors, rating scales

## Abstract

An increasing number of young people are becoming socially and economically marginalized in Japan under economic stagnation and pressures to be more globally competitive in a post-industrial economy. The phenomena of NEET/Hikikomori (occupational/social withdrawal) have attracted global attention in recent years. Though the behavioral symptoms of NEET and Hikikomori can be differentiated, some commonalities in psychological features can be found. Specifically, we believe that both NEET and Hikikomori show psychological tendencies that deviate from those governed by mainstream cultural attitudes, values, and behaviors, with the difference between NEET and Hikikomori being largely a matter of degree. In this study, we developed a NEET-Hikikomori Risk Factors (NHR) scale that treats NEET/Hikikomori not as a set of distinct diagnoses, but as a spectrum of psychological tendencies associated with the risk of being marginalized in society. Based on this idea, we identified three related risk factors in our NHR spectrum scale: (1) *Freeter lifestyle preference*, which in Japan refers to the tendency to consciously choose to not work despite job availabilities, (2) a lack of self-competence, and (3) having unclear ambitions for the future (Study 1). Study 2 investigated and confirmed the validity of the scale by examining NHR differences between occupational groups. The results suggested that NHR is related to psychological tendencies common in the marginalized segments of society. The relationship between these psychological tendencies and actually becoming marginalized across cultures is discussed.

## NEET and Hikikomori and psychological spectra

Since entering the twenty-first century, an increasing proportion of young Japanese have become socially/culturally marginalized. Specifically, many of them are emerging as “NEETs (Not in Employment Education or Training; Genda, 2005)” or “Hikikomori” (socially withdrawn/isolated). The Ministry of Health, Labor and Welfare defined NEET as “people who are not employed, not in school, not a homemaker, and not seeking a job” and Hikikomori as “those who are neither in work nor school, do not have social interactions and are socially withdrawn for more than 6 months.”

Based on these classification criteria, the Cabinet Office of the Japanese Government (2010) estimated that there were about 700,000 Hikikomori. Also, a recent epidemiological study confirmed a life-time prevalence rate of about 1.2%, or a little over 1.5 million individuals in the Japanese population, who are or have been Hikikomori, with half of them not having a comorbid DSM-IV diagnosis (Koyama et al., 2010).

Possible triggers of Hikikomori might include school absenteeism (*futouko*u) or job insecurity. In fact, both the numbers of Hikikomori and school refusers have been increasing (Jones, 2006) for the obvious reason that it becomes increasingly difficult to reintegrate into society the longer one remains socially withdrawn. Consequently, it is not unusual to find a Hikikomori who has been withdrawn for over a decade (Sakai et al., 2011).

Zielenziger (2006) has suggested that this is a cultural syndrome specific to the Japanese context, since the number of Hikikomori (about 1.2% of the population; Koyama et al., 2010) in Japanese society is not insignificant. However, some researchers have reported cases of NEETs or Hikikomori in other countries such as the UK, Korea, Italy, Spain, and France (Kato et al., 2012; Pilz et al., 2013), although the prevalence and average length of withdrawal are not fully known in these other countries.

Though there are distinct differences in the behavioral symptoms of NEET and Hikikomori, some commonalities in psychological tendencies can be found as well. First, some literature has suggested that autistic tendencies might affect both NEETs and Hikikomori (e.g., Hoshino, 2011). Second, they are usually young adults under 40, who are supposed to be full of energy in school or at work, but instead are not engaged in many social interactions with other people, including with their own family members. Third, they are not able to secure a job or sense of belonging (Brinton, 2011). Some of them are “Freeters^1^” who are perpetually engaged in low-skill part-time employment. Others are Hikikomori or shut-ins. In sum, both NEET and Hikikomori show a tendency to deviate from mainstream cultural attitudes and values. The spectrum-based approach is found in other psychological disorders such as autistic spectrum disorder. The spectrum approach is useful for identifying the risk for people who have not been diagnosed with a psychopathology (Koyama et al., 2010).

Thus, as the aims of this study are to measure the tendencies to deviate from the cultural mainstream rather than to identify the detailed behavioral patterns found in NEET and Hikikomori, we employed the psychological commonality approach rather than the behavioral categorical differentiation approach. While the differential approach may have advantages for finding appropriate interventions that are suitable for behaviors diagnosed as NEET or Hikikomori, the psychological commonality approach has the advantage of allowing us to analyze the mechanisms and processes involved in the social marginalization of youths.

### Marginalization risk and its consequences

In a series of empirical and theoretical studies, Norasakkunkit and his colleagues argued that such marginalization has resulted from economic and social structural changes in post-industrialized societies (Norasakkunkit and Uchida, 2011, 2012; Toivonen et al., 2011). Furthermore, Ogihara and Uchida (2014) suggested that globalization has created a pressure toward increasing individualism, especially the kind that seems to be practiced in the United States (also see Hamamura, 2012). Rising individualism seems to be a feature of all post-industrial societies (Arnett, 2011), including the United States (Twenge et al., 2012), due to increased competition at the individual level (as opposed to the intergroup level; Yuki and Brewer, 2014), declining blue collar jobs and increasing white collar jobs (Grossmann and Varnum, 2015), as well as globalization's individualistic ideas and ideals (Hamamura, 2012; Ogihara et al., 2015). However, individualism is not necessarily the same everywhere. Whereas, American individualism is deeply rooted in tradition and religious moral foundations, such as the Protestant work ethic and Puritan ideals (Weber, 1920; Bellah et al., 2007), any imported version of individualism is likely to be stripped of such rich foundations and may lead to feelings of isolation and unhappiness, or even to irresponsible, reckless behavior. Similarly, any imported version of collectivism that is stripped of its traditional and moral foundations, such as Confucianism or Buddhism, may appear to be unsophisticated group think or “adolescent cliquishness.” Thus, individualism in Japan is not likely to be identical to that of Western societies, no more than American collectivism is likely to be identical to East Asian collectivism. Consequently, increasing individualism in Japan without the strong foundations that American individualism has been built on may very well contribute to confusion for many individuals who try to become “individualistic,” especially if they use it as an excuse to avoid interpersonal responsibilities and obligations, or to develop interpersonal skills that will let them become attuned to their in-group members (Ogihara and Uchida, 2014).

Indeed, it has been argued that such cultural change can potentially have a negative impact on mental health, especially among young people, since it would affect their own cultural competence to function successfully within their own society (Toivonen et al., 2011), as well as their sense of continuity in their self-concept (Chandler et al., 2003).

Though youth problems such as NEET and Hikikomori have attracted much global attention, the contributing factors and psychological tendencies are not well understood. Furthermore, such cultural marginalization is not simply a categorical diagnosis but is more likely to represent a psychological spectrum from simply not being engaged in occupational activities (being Freeter or NEET) to completely withdrawing from all social interactions (being Hikikomori). In this paper, we developed (Study 1) and validated (Study 2) a NEET-Hikikomori risk spectrum measure and discussed its psychological meanings and consequences.

### Cultural standards and cultural change

In the cultural psychological literature, it has been argued that Japanese people are more likely to have an interdependent selves, due to living in a more collectivistically oriented cultural environment (Markus and Kitayama, 1991, 2010) that may be rooted in an agricultural social structure (Nisbett, 2003). Scientific data have supported this thesis, especially those that examine cross-cultural studies comparing Japanese and North Americans. In contrast to the North Americans' “independent” construal of self, Japanese are more likely to be oriented toward harmonious social relationships in emotion or subjective well-being (Kitayama et al., 2006; Uchida et al., 2008), motivation (Heine et al., 2001; Kitayama et al., 2004), cognition (Masuda and Nisbett, 2001), and even mental anguish (Norasakkunkit et al., 2012; Sato et al., 2014). All of these studies have investigated dominant psychological tendencies in each culture. While such studies have represented what scholars have been discussing in terms of stable cultural differences for the last 20 years (e.g., Markus and Kitayama, 1991; Triandis, 1995; Shweder, 1996), now is the time to think about the more dynamic psychological mechanisms within a culture that result from cultural changes that are occurring due, in part, to forces exerted from outside the culture, such as globalization. The NEET-Hikikomori spectrum may very well represent a consequence of such dynamic forces.

While it is tempting to think of the NEET/Hikikomori spectrum as a culture-bound syndrome, it is perhaps not comparable to other culture-bound syndromes such as *Taijin Kyofusho* (Kasahara, 2005), because the emphasis on the *spectrum* aspect of NEET/Hikikomori suggests that our goal is not to come up with a checklist of diagnoses for a discrete disorder or syndrome and recommend a treatment. Instead, our goal is to measure the psychological *tendencies* within the spectrum that represent degrees of marginalization that are rooted in the larger, dynamic cultural and global forces associated with various occupational segments of the population in a globalizing, postindustrial society like Japan.

### Current paper

Existing approaches in identifying NEET/Hikikomori have emphasized demographic/behavioral characteristics, such as “staying home for more than 6 months” and “not being in school or a job.” Such an approach fails to capture the potential or risk that is associated with becoming NEET or Hikikomori. Again, our goal is to assess NEET/Hikikomori as a psychological risk spectrum to be measured. While the existing literature tends to treat NEET and Hikikomori as distinct, we are interested in examining their commonalities based on the common attitudes and values held by both NEETs and Hikikomori that suggest a deviance from the mainstream cultural attitudes and values.

The first part of this paper discusses how we developed the NEET-Hikikomori Risk scale (NHR scale) to measure the NEET-Hikikomori risk spectrum among youth. In the second study, we analyzed a large nation-wide survey to: (1) investigate the theoretical validity of the NHR scale relative to the government's nominal classification of NEETs and Hikikomori, and (2) investigate whether the NHR is more strongly associated with outcomes that the government's nominal classification of NEET and Hikikomori have been associated with. These include subjective well-being, subjective health, job satisfaction, and quality of social relationships (outside the family).

## Study 1: developing the NH risk scale

We developed the NEET/Hikikomori Risk (NHR) scale to measure the potential risk of becoming NH among young people. Previous studies have only examined NEET and Hikikomori as distinct categories with very few exceptions (Watanabe et al., 2010). With our scale, we identify psychological risk factors for NH for young people who are currently in some form of employment (including university students). Thus, in this study we first identify the shared behavioral/psychological patterns of NH based on previous research and devise scaled items to measure NHR tendencies.

### Item selection

In the first stage, we sampled and compiled as many attitudes/opinions/values/behavioral patterns/psychological patterns as possible that have been commonly reported by NEET and Hikikomori according to nine randomly selected full volume books written by sociologists, psychiatrists, and psychologists that both had “NEET,” “Freeter,” or “Hikikomori” in the title and also discussed behavioral/psychological features. All the books were published before June 2008 (see Appendix 1 in Supplementary Material). First, we randomly selected six of the nine books, and searched these for clear descriptions and descriptions of NH, associated behaviors and psychological attitudes, as well as other items that could potentially be included in our survey. Three coders identified 213 relevant items. We coded each item into one of nine categories: (1) the reason for not being employed (e.g., “cannot find a job that fits the with skill/ability/knowledge level”; Inui et al., 2006), (2) attitudes toward working (e.g., “cannot find a reason to work,” Inui et al., 2006), (3) attitudes toward academic training (e.g., “it is not important to study hard in order to get a job in the future,” Honda et al., 2006), (4) attitudes toward society (e.g., “Society should provide opportunities to do other things besides working at a job,” Inui et al., 2006), (5) self-competence and goal orientation (e.g., “I do not have basic skills,” Taromaru, 2006), (6) relationships (e.g., “There is nobody whose support I can count on when times are tough,” Taromaru, 2006), (7) behavior (e.g., “don't like to go outside much,” Honda et al., 2006) (8) family (e.g., “There are conflicts within the family,” Honda et al., 2006), and (9) valuing of cognition, emotion (e.g., “our reliance on a hierarchical social system is incompatible with reality,” Honda et al., 2006).

In the second stage, we examined the remaining three books and coded any description that could fall into one of the nine categories discussed above. 89.1% of descriptions were categorized within the nine categories and 70.3% of the descriptions overlapped with 213 of the above items in their meanings.

In the final stage, four coders collapsed the 213 items into 53 items (including 21 negatively keyed items) by identifying similar descriptions and combining them into a single item. These 53 items constituted our pre-NH scale.

### Procedure

Participants were 66 (34 males and 32 females) Japanese undergraduate students. Their age ranged from 18 to 23. We asked them to evaluate themselves on 53 items on a seven-point Likert scale ranging from “Completely Disagree” to “Completely Agree.” The survey study was conducted carefully following the Japanese Psychological Association ethical guidelines.

### Results and discussion

We ran a Factor analysis with a maximum likelihood estimation model and found that the loading score had decreased by the third factor (6.33, 4.31, 2.89) but it was unchanged after the fourth factor (2.07, 1.86) so we settled on three factors. We checked the factor loadings with promax rotation. Factor 1 was labeled “Freeter Lifestyle Preference,” which refers to the likelihood of becoming a “Freeter” who consciously chooses to not work despite opportunities and job availabilities. An example of an item from this factor is, “I don't think it is necessary to find a job immediately.” Factor 2 has to do with a “Lack of Self Competence” (i.e., not feeling competent at accomplishing interdependent cultural tasks). An example of an item from this factor is, “My social skills are low, and I am not good at relating to others.” The last factor has to do with an “Unclear Ambition for the Future” (i.e., having unclear or unrealistic goals for what they want to do in the future.” An example of an item from this factor is, “I don't quite know what I want to do in the future.” We chose only the 35 items (17 from Factor 1, 13 from Factor 2, 5 from Factor 3) that had over 0.35 factor loadings and did not have a high loading score (above 0.30) on more than two factors. Thirty-five items (17 from Factor 1, 13 from Factor 2, 5 from Factor 3) were chosen. We further deleted eight items (3 from Factor 1, 2 from Factor 2, 3 from Factor 3) to maximize Cronbach's α within each factor. The results revealed 27 meaningful survey items (14 from Factor 1, 11 from Factor 2, 2 from Factor 3) which measured the risk of becoming NH (α = 0.79−0.83) (Table 1). These three risk factors represent at least three different types of NH subcategories. Although these subcategories are relatively distinct from each other, a reliability analysis also revealed that an overall score that combined all items could represent an overall NHR as indicated by a Cronbach's alpha of 0.82. Those who scored in the top 15% on the NH overall score were classified as relatively high risk, assuming that the scale will be used for dichotomizing purposes. The correlation between Factor 1 and Factor 2 was 0.35 (*p* < 0.001), between Factor 1 and Factor 3 was 0.32 (*p* < 0.002) and between Factor 2 and Factor 3 was 0.42 (*p* < 0.001).

**Table 1:** **NEET Hikikomori Risk (NHR) scale**.

**Item**			**I**	**II**	**III**
**FACTOR 1: FREETER LIFESTYLE PREFERENCE (α = 0.83)**					
	I don't think it is necessary to find a job immediately.	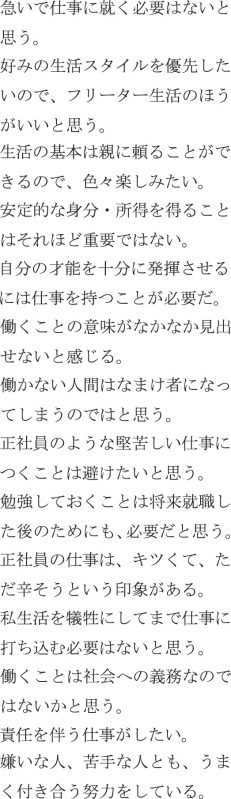	0.67	0.00	−0.05
	Since I want to prioritize my own preferred lifestyle, it would be better to lead a part time freelance lifestyle (than to get a formal full time job).		0.66	0.11	0.10
	Since I can rely on my parents to provide for my basic needs, I want to try to have all kinds of fun.		0.66	−0.14	−0.01
	Obtaining stability and a high salary is not that important to me.		0.64	−0.08	−0.11
#	I think it is necessary to have a job in order to sufficiently be able to fulfill one's talents.		0.58	0.08	−0.28
	I can not find meaning in work.		0.52	0.22	−15
#	I think that a person who does not work will become lazy.		0.52	−0.26	−0.06
	I would like to avoid getting a job in a very formal kind of full time position.		0.51	0.14	0.01
#	It is necessary to study now for what may come after I get a job.		0.48	−0.18	−0.03
	My impression of the work of a regular full-time employee is that it is intense and just seems harsh.		0.40	0.14	0.09
	I don't think it is necessary to pour myself into my work to the extent of sacrificing my private life.		0.39	0.16	0.11
#	I think that to work is to fulfill one's duty to society.		0.37	−0.15	0.06
#	I would like to do work that comes with responsibilities.		0.37	0.10	0.07
#	Even if it is someone I don't like or someone who is difficult to deal with, I make an effort to get along with people.		0.37	0.17	−0.02
**FACTOR 2: LACK OF SELF COMPETENCE (α = 0.83)**					
	I feel that communicating with others is hopelessly difficult for me.	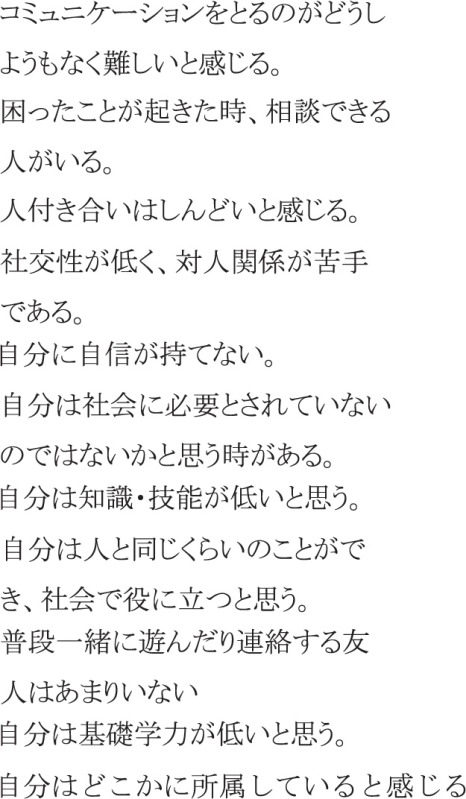	−0.11	0.77	0.08
#	When I have some trouble to deal with, I have someone I can talk to.		−0.08	0.64	−0.09
	Mingling with others is exhausting for me.		−0.04	0.61	0.03
	My social skills are low, and I am not good at relating to others.		−0.10	0.61	0.03
	I don't have confidence in myself.		−0.16	0.54	0.04
	There are times when I think that I am not needed by society.		0.18	0.53	0.23
	I think that my basic abilities are low.		0.08	0.52	0.27
#	I can do just as much as anybody else and I think I will be useful to society.		0.25	0.50	−0.03
	I don't have too many friends who I can go out with or call.		−0.22	0.44	−0.16
	I think that my knowledge and skills are at low levels.		0.04	0.41	0.18
#	I feel that I belong somewhere.		−0.14	0.40	−0.06
**FACTOR 3: UNCLEAR AMBITION FOR THE FUTURE (α = 0.79)**					
	I don't quite know what I want to do in the future.	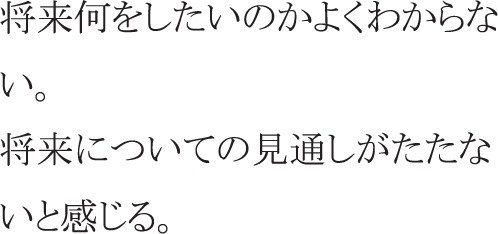	0.14	−0.21	0.81
	I feel like I don't have a clear future prospect.		0.04	−0.04	0.79

*# Negatively keyed items requiring reverse scoring*.

In sum, Study 1 suggested that the risk factor of NEET-Hikikomori contains three factors, each related to an attitude toward career, self, and the future. All these tendencies were psychological and thus were measurable, even for those not officially classified as NEET or Hikikomori. In the following studies, we checked the validity of these factors.

## Study 2

Study 2 analyzed a large nationwide survey of young Japanese people (aged 20 to 39) conducted by the Japanese Government (Cabinet Office, Government of Japan). The purpose of this study was to: (1) examine the association between “the classification of NEET and Hikikomori” employed by the Japanese government and the NHR spectrum as measured by our scale, and (2) examine how each of the variables above (i.e., classification of NEET, classification of Hikikomori, and NHR scale) was related to socioeconomic status (level of education, income, and employment status) as well as well-being related indices (general well-being, subjective health condition, job satisfaction, and close relationships).

### Method

The survey data were collected via the Internet as part of a large national survey conducted by the Economic and Social Research Institute of the Cabinet Office, Government of Japan for the purpose of a secondary analysis. The survey research was conducted as a panel data set obtained in December 2010 and March 2011. All participants were aged between 20 and 39 years. The total number of survey respondents was 10,744, but we excluded students and homemakers, resulting in a sample of 7,725 (62.32% male, 32.22% female) participants. 4.38% of the participants were aged between 20 and 24 years old, 19.50% between 25 and 29 years old, 30.56% between 30 and 34 years old, and 45.56% between 35 and 39 years old.

### Measures

#### NHR scale

Same as Study 1. Cronbach's alpha in this sample is 0.88, 0.75, 0.88, and 0.68 for overall NHR score, Factor 1, Factor 2, and Factor 3, respectively.

#### Classification criteria of NEET and Hikikomori

The Ministry of Health, Labor and Welfare defined NEETs as “people who are not employed, not in school, not a homemaker, and not seeking a job” and defined Hikikomori as “those who are neither in work nor school, do not have social interactions and are socially withdrawn for more than 6 months.” To assess the government's classification of NEET and Hikikomori, we relied on the participants' current occupational status and other activities. These included whether they are currently in a job or seeking a job, as well as the frequency with which they went outside the home. Based on these criteria, there were 200 people who were categorized as NEET (1.86% of the total sample of 10,744) and this prevalence was consistent with what the government has reported (around 2% in 2011). Amongst the 200 NEETs, 114 of them also met the criteria for Hikikomori (1.06% of the total sample of 10,744 participants) as they had not been out of their home, with few exceptions (such as going to convenience stores), for more than 6 months. The prevalence of Hikikomori in this sample was consistent with the 1.2% rate for those between the ages of 20 and 49 reported in a previous epidemiological study (Koyama et al., 2010).

#### Employment status

Participants were asked to state whether they were: (1) full-time employees, (2) part-time employees, or (3) not employed. Those who were not in employment were asked whether they were seeking a job or not. If they were in the third category and not seeking job, they were classified as NEET, but if they were seeking a job, they were classified as “unemployed.”

#### Socioeconomic status

The participants were asked about their highest educational attainment and how much income they earned in the year of 2010. 1.96% attained only a junior high school education, 20.58% attained only a high school education, 22.13% were vocational college or 2-year college graduates, 46.06% were university graduates, and 9.27% had attained either a Master's degree or Ph.D. The average household income was 4,210,000 yen (*SD* = 8, 772, 922), which is approximately US $42,000 (*SD* = $87,000).

#### Well-being

We asked participants to rate their “general well-being” on the basis of a question used in the European Social Survey: “To what extent are you happy?” (0 = very unhappy, 10 = very happy; Inglehart, 1997). Participants also reported their subjective health condition (“to what extent do you feel you are in good health?”) on a five point scale (1 = not healthy at all, 5 = very healthy). They also reported their job satisfaction (“to what extent are you satisfied with your job?”; 1 = not satisfied at all, 4 = very satisfied).

#### Close relationships within the community

One item asked “to what extent do you interact with neighbors in your community?” (0 = not at all, 1 = exchange greetings, 2 = have conversations with someone on a daily basis 3 = have close relationships or have someone who could help me when I'm in trouble).

### Results and discussion

#### Relationships between NHR scale and the government classification of NEET and Hikikomori

Those who were classified as NEET according to the government's criteria scored significantly higher on NHR (*M* = 4.45) than those who were not classified as NEET (workers) (*M* = 3.55), *t*_(7723)_ = 17.12, *p* < 0.0001. Significant differences were also found in three subcategories (Factor 1: *M* = 3.92 vs. 3.24, *t* = 12.84, Factor 2: *M* = 4.98 vs. 3.81, *t* = 15.87, Factor 3: *M* = 5.29 vs. 4.26, *t* = 10.55, all *p*s < 0.0001).

**Figure 1:**
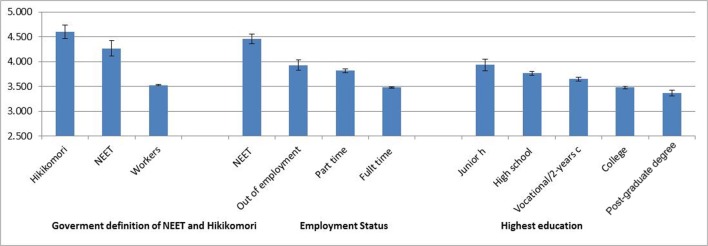
**NHR scores across demographic features in large sample (Study 2)**. Bars show 95% confidence intervals.

A closer examination, using a UNIVariate-ANOVA on NHR by three categories using the government's classification (1 = NEET but not Hikikomori, 2 = Hikikomori, 0 = workers) revealed that the main effect was significant [*F*_(2, 7722)_ = 151.53, *p* < 0.0001, η_*p*_^2^ = 0.040], with Hikikomori scoring the highest on NHR (*M* = 4.59), followed by NEETs (*M* = 4.27), followed by working people (*M* = 3.55), and all of these groups were significantly different from each other (*p*s < 0.05, see Figure 1). This pattern was replicated when Factors 2 and 3 were examined separately [*F*_(2, 7722)_ = 136.07 and 109.01, *p*s < 0.0001, η_*p*_^2^ = 0.03 and 02], respectively. Specifically, scores were higher for Hikikomori, followed by NEETs, followed by workers, and all of these groups were significantly different from each other (*p*s < 0.05; see Table 2). As for Factor 1, there was a significant main effect [*F*_(2, 7722)_ = 82.60, *p* < 0.0001, η_*p*_^2^ = 0.02], but there were no significant differences between NEETs and Hikikomori, although both of their scores were higher (*p*s < 0.05) than those of working people.

**Table 2:** **Mean scores across demographic factors (Study 2)**.

		**Factor 1 Freeter lifestyle preference**	**Factor 2 Lack of self-competence**	**Factor 3 Unclear ambition for the future**
		**Mean *(SD)***		
Government classification	Workers (*N* = 7525)	3.25 (0.73)	3.81 (1.03)	4.26 (1.37)
	NEET (*N* = 86)	3.89 (0.80)	4.61 (1.14)	5.05 (1.41)
	Hikikomori (*N* = 114)	3.95 (0.80)	5.26 (1.16)	5.48 (1.60)
	Between sample difference in each factor *F*	82.60^***^, η_*p*_^2^ = 0.02	136.07^***^, η_*p*_^2^ = 0.03	109.01^***^, η_*p*_^2^ = 0.02
Job status	Full-time employee (*N* = 5879)	3.19 (0.73)	3.73 (1.00)	4.15 (1.33)
	Part-time employee (*N* = 1242)	3.49 (0.72)	4.08 (1.04)	4.67 (1.38)
	Unemployed (but not NEET) (*N* = 335)	3.37 (0.74)	4.47 (1.12)	4.88 (1.58)
	NEET (*N* = 200)	3.92 (0.80)	4.98 (1.19)	5.30 (1.54)
	Between sample difference in each factor *F*	119.81^***^, η_*p*_^2^ = 0.04	159.77^***^, η_*p*_^2^ = 0.06	107.20^***^, η_*p*_^2^ = 0.04
Education level	Post-graduate degree (*N* = 713)	3.10 (0.76)	3.60 (0.96)	3.92 (1.36)
	College (*N* = 3543)	3.20 (0.75)	3.70 (1.03)	4.19 (1.36)
	Vocational/2-year college (*N* = 1702)	3.32 (0.70)	3.93 (1.00)	4.41 (1.34)
	High school (*N* = 1583)	3.39 (0.73)	4.11 (1.08)	4.51 (1.40)
	Junior high school (*N* = 151)	3.50 (0.83)	4.39 (1.17)	4.57 (1.41)
		31.95^***^, η_*p*_^2^ = 0.02	67.25^***^, η_*p*_^2^ = 0.03	33.14^***^, η_*p*_^2^ = 0.02

*** *p < 0.001*.

#### NHR and socio-economic status

We defined job status as full-time employee (*N* = 5976), part-time employee (*N* = 1359), unemployed (but not NEET since they were seeking a job; *N* = 190) and NEETs who were not seeking job (*N* = 200) and conducted an ANOVA (job status × gender) on the NHR score, since how much job status matters would depend on gender, given that the social pressure for being in a secure job is stronger for males than for females in contemporary Japan. There was a main effect of job status [*F*_(3, 7717)_ = 181.78, *p* < 0.0001, η_*p*_^2^ = 0.07] showing that the NEETs scored the highest (*M* = 4.43), followed by the unemployed (*M* = 3.93), part-time workers (*M* = 3.82), and full-time employees (*M* = 3.47), with all groups being significantly different from each other (see Figure 1). This result suggested that not only NEETs, but also the unemployed who are seeking jobs and part-time workers, are at risk of becoming NEET or Hikikomori relative to full time employees. There was no significant gender effect [*F*_(1, 7717)_ = 3.14, *p* < 0.08, η_*p*_^2^ = 0.00] nor any interaction effects [*F*_(1, 7717)_ = 1.25, ns, η_*p*_^2^ = 0.00] (see Figure 1). The same main effect of job status was found for Factors 2 and 3 [*F*_(2, 7717)_ = 152.45 and 91.85, *p*s < 0.0001, η_*p*_^2^ = 0.06 and 0.04, wherein the NEETs scored the highest, followed by the unemployed who are seeking a job, part-time workers, and full-time employees, with all groups being significantly different from each other (*p*s < 0.05, see Table 2). As for Factor 1, there was also a significant main effect of job status [*F*_(2, 7717)_ = 102.56, *p* < 0.0001, η_*p*_^2^ = 0.04], where NEETs scored the highest. However, this time part-time workers scored second highest, followed by the unemployed who are seeking a job, followed by full-time employees, with all groups being significantly different from each other (*p*s < 0.05, see Table 2). No gender effect (both main effect and interaction effect) was not found for any of the three factors, except for a significant interaction effect of job status by gender on Factor 3 [*F*_(3, 7717)_ = 3.60, *p* < 0.02, η_*p*_^2^ = 0.001], although the effect size was too small to warrant further discussion.

As for level of education, we again conducted a UNI-variate ANOVA on NHR score. The main effect of education was [*F*_(4, 7687)_ = 69.12, *p* < 0.0001, η_*p*_^2^ = 0.04], showing that those who attained only a junior high school education scored the highest (*M* = 3.94), followed by those who attained only a high school education (*M* = 3.77), followed by vocational school graduates or 2-year college graduates (*M* = 3.65), followed by college graduates (*M* = 3.48), followed by those with a Master's Degree or Ph.D. (*M* = 3.36; see Figure 1). The same pattern emerged for Factor 2 [*F*_(4, 7687)_ = 67.25, *p* < 0.0001, η_*p*_^2^ = 0.03], with all groups being significantly different (*p*s < 0.05, see Table 2). As for Factor 1, there was also a significant main effect [*F*_(4, 7687)_ = 31.95, *p* < 0.0001, η_*p*_^2^ = 0.02], and junior high and high school graduates scored the highest (they were not significantly different from each other) followed by vocational school graduates or 2-year college graduates, college graduates and those with a Master's Degree or Ph.D. (see Table 2). There was a significant main effect for Factor 3 as well [*F*_(4, 7687)_ = 33.14, *p* < 0.0001, η_*p*_^2^ = 0.02], with junior high, high school, and vocational college or 2-year college graduates scoring the highest (they were not significantly different), followed by college graduates, followed by those with a Master's Degree or Ph.D. (see Table 2).

Employment status and socio-economic status were related to each other (*r* = 0.19, *p* < 0.001). In order to identify which was the better predictor of NHR, we conducted a regression analysis on NHR by employment status and socioeconomic status. The result revealed that each factor independently predicted NHR (β = 0.24 and 0.14, for employment and socioeconomic status, respectively, *p*s < 0.0001, *R*^2^ = 0.09).

#### Relationships between NHR, government classification of NEET, government classification Hikikomori, and well-being

General well-being was significantly correlated with NHR (*r* = −0.45, *p* < 0.0001), suggesting that those who were higher in NHR scored lower on subjective well-being (*r*s = -0.23, −0.49, and −0.46 for Factor 1, Factor 2, and Factor 3, respectively, all *p*s < 0.0001), as expected. When we regressed general well-being on NHR and the government's nominal classification of NEET/worker (1 or 0), NHR was more strongly associated with well-being (β = −0.44) than the nominal classification of NEET/worker (β = −0.06), *R*^2^ = 0.21^2^. Self-reported health status was significantly correlated with NHR (*r* = −0.30, *p* < 0.0001), showing that those who were higher in NHR tended to self-report lower health status (*r*s = −0.17, −0.31, and −0.24 for Factor 1, Factor 2, and Factor 3, respectively, all *p*s < 0.0001). When we ran a regression analysis on subjective health condition as the dependent variable and NHR and government classification of NEET (1 or 0) as independent variables, NHR was more strongly associated with subjective health (β = −0.27) than the nominal classification of NEET (β = −0.12), *R*^2^ = 0.10.

Job satisfaction was also significantly correlated with NHR (*r* = −0.32, *p* < 0.0001), suggesting that those who were higher in NHR tended to have lower job satisfaction (*r*s = −0.20, −0.30, and −0.35 for Factor 1, Factor 2, and Factor 3, respectively, all *p*s < 0.0001). When we regressed job satisfaction on NHR and the nominal classification of NEET/worker, NHR was more strongly associated with job satisfaction (β = −0.32) than the nominal classification of NEET/was (β = 0.01), *R*^2^ = 0.10. The result was not any different when we replaced the nominal classification of NEET/worker with employment status (1 = full-time employee, 2 = part-time employee, 3 = unemployed, 4 = NEET) (job status showed lower β; −0.08). Close relationships within the community were also negatively correlated with NHR (*r* = −0.22, *p* < 0.0001), suggesting that those who scored higher on NHR had fewer intimate relationships (*r*s = −0.12, −0.24, and −0.16 for Factor 1, Factor 2, and Factor 3, respectively, all *p*s < 0.0001). Regressing close relationships within the community on NHR and the nominal classification of NEET/worker (1 or 0) suggested that NHR was more strongly associated with subjective health (β = −0.21) than the nominal classification of NEET/worker (β = −0.05), *R*^2^ = 0.22.

In summary, NHR was theoretically validated vis-à-vis the government's classification of NEET and Hikikomori, and was also shown as capable of discriminating between these groups and lesser degrees of marginalization in Japanese society. Moreover, the NHR is more strongly and inversely associated with general well-being, self-reported health status, job satisfaction, and close relationships within Japanese society than the nominal classifications of NEET, Hikikomori, and workers. Furthermore, NHR was also found to be higher for those with lower educational attainment and those with less stable jobs. Thus, NHR was positively associated with increasing degrees of marginalization as a function of educational level or occupational status.

## General discussion

We tried to define NEET and Hikikomori as a spectrum of psychological tendencies associated with degrees of marginalization in Japanese society, including the extreme end of this spectrum that is associated with the segment of the Japanese population who has become cultural dropouts. Based on this idea, we identified psychological risk factors and developed measurements for them. The advantage of treating the NH phenomena as part of the spectrum of psychological tendencies associated with degrees of marginalization is that it allows us to analyze the mechanism and processes of the marginalization of youth in society. Furthermore, as Study 2 suggested, the psychological spectrum approach was more strongly associated with subjective well-being than the classification approach. This means that NHR is prevalent even among regular employees who are not classified as NEET or Hikikomori, and that NHR is not necessarily strongly correlated with socio-economic status (SES) even though it is significantly correlated with SES. In the end, this NHR spectrum taps into psychological tendencies that are associated with marginalization rather than capturing actual marginalization *per se*. Therefore, another advantage of the spectrum approach is that it can identify behavioral risks associated with potentially becoming NH in the future and can therefore contribute to identifying who can benefit from early interventions that help to prevent marginalization in their society. Finally, a more general advantage of taking a spectrum approach is that it can be applied not only to NHR but to other psychological disorders, such as depression. This advantage provides a rationale for developing a more fine-tuned psychological spectrum-based measure rather than simply a checklist of symptoms to classify disorders.

One potential criticism of the NHR scale we developed is that the NHR spectrum may simply be tapping into tendencies of already-known psychological disorders such as depression and is therefore not necessarily measuring tendencies that are distinct to NH. Readers may even notice some symptom overlap between Hikikomori tendencies and depressive tendencies, such as annhedonia. Indeed, previous studies have found that the only DSM psychological disorder that is reliably associated with the NEET/Hikikomori tendency is the depressive tendency. However, even Hikikomori, which lies at the extremely pathological end of the NHR spectrum, is associated with only mild-to-moderate levels of depression and not clinical levels of depression (Koyama et al., 2010; Norasakkunkit and Uchida, 2010). Furthermore, most severely depressed people in Japan are not Hikikomori, nor are most Hikikomori severely depressed. Thus, we agree with the originator of the term “Hikikomori,” Saito (1998), that Hikikomori is distinct from depression or any other known psychological DSM disorder, despite some psychiatrists (e.g., Saito, 2010) who suggest otherwise (see also Norasakkunkit and Uchida, 2014 for a more elaborate test and discussion of this issue).

From the scale development study (Study 1) and the large sample survey study (Study 2), we found that NEET-Hikikomori risk can be measured as psychological tendencies that include even people who were not currently classified as NEET or Hikikomori. We successfully developed a 27-item NHR scale (Study 1) and established its reliability and validity with respect to having NHR scores correspond with varying degrees of marginalized strata in a large-scale, nationwide Japanese sample (Study 2).

In terms of the content of the NHR scale, we identified three risk factors associated with attitudes related to risk of becoming NEET and Hikikomori. Namely, “Freeter lifestyle preference,” “Lack of self-competence,” and “Unclear ambition for the future.”

The first factor, “Freeter lifestyle preference,” represents the resistance to conforming to the cultural standard for becoming a socially sanctioned “adult” in society. Factor 1 items are mostly about harboring attitudes that deviate from occupations that conform to conventional ideas of what constitutes a full member of society. The score of Factor 1 was higher for part-time employees rather than those who were unemployed (Study 2), suggesting that the Freeter lifestyle is actually more about engaging in non-traditional and unstable work than about not being engaged in work at all. Other studies also suggest culturally deviant motivational styles among those who score high on the NHR scale. For example, those who score high on the NHR scale were much less likely to conform to their in-group's behaviors than those who do not score high on the NHR scale (Norasakkunkit and Uchida, 2014).

The second factor, “Lack of self-competence,” includes confidence in social skills and academic/working skills. People with low self-competence would feel anxiety or even fear when encountering obstacles, such as might occur during job-hunting or in interpersonal conflicts. Norasakkunkit and Uchida (2011) also provided behavioral evidence that people with high NHR scores in Japan were not motivated to work harder in response to a failure experience, even though becoming motivated to work harder after a failure experience is the predominant motivational pattern in Japan; Heine et al., 2001). Also, those who score high on the NHR scale have been found to be much less likely to cease exploring their identity in order to actively search for a job in their final two years of college than those who do not score high on the NHR scale (Norasakkunkit et al., 2011). Thus, the deviation from dominant motivation patterns among those at risk of becoming NH may be partly due to a fear of facing challenges and obstacles, and partly due to a lack of willingness to conform to culturally sanctioned behaviors (Norasakkunkit and Uchida, 2014).

Some of the NPOs that try to rehabilitate NEETs and Hikikomori in Japan have intervened at the level of guiding parents of NEET/Hikikomori by suggesting effective ways to show appreciation and admiration of their children (e.g., Sakai et al., 2011). Future studies that investigate developmental issues, including experiences in childhood, can focus on parent-child interactions that are associated with developing self-competence and a motivation to be in the society.

The third factor, “Unclear ambitions for the future,” is about whether individuals hold a clear vision on what they might want to do in the future. Currently, some studies (e.g., Arnett, 2004; Newman, 2012) suggest that it is taking longer for individuals to feel like they have become an adult (usually into their late 20s) in post-industrialized societies like Japan and the United States than in the past (usually right after adolescence). In such societies, not only NEETs and Hikikomori, but also young working people and college students, often feel that their futures are unclear given that the score of Factor 3 was higher than the other two factors across samples.

Indeed, Study 2 suggested that Factor 3 was the highest score and Factor 1 was the lowest in every segment of the population examined in our study. While the behaviors and attitudes of NEETs that have been highlighted most prominently, especially by the media, are those that are measured by Factor 1 (“Freeter lifestyle preference,”) this notion is somewhat misleading because it suggests that NEETs are rugged individualists who are resisting social conformity. However, our data suggests that NEETs and Hikikomori are characterized more by low self-competence and lacking clear ambitions for the future.

However, one limitation of Study 1 was that the scale development procedure did not include a confirmatory factor analysis, which would have confirmed the validity of the three factor designs. To overcome this limitation in Study 1, Study 2 examined and revealed the NHR scale's theoretical, concurrent validity, and discriminant validities. Overall NHR scores were higher for NEET, Hikikomori, and the unemployed group than for working people (Study 2). Part-time workers scored significantly higher on NHR than full-time employees, and those with lower educational attainment scored significantly higher on NHR than those with higher educational attainment, suggesting that the NH tendencies are also related to socioeconomic factors and sociological markers of marginalization. Furthermore, Study 2 showed that the NHR scale discriminated between all the occupational groups (such as NEET, unemployed, and part-time workers) and found that the NHR scores were better predictors of lower well-being, lower job satisfaction, and fewer close relationships than was the government nominal classification of NEET. Sometimes NEET and Hikikomori are perceived as resulting from overprotection by upper-middle class parents who can afford to pay their children's living expenses, but our results suggest that this is largely not the case. However, we should be cautious about making claims about causal mechanisms, which might be bidirectional. Lower job security or lower education level might be a cause of NHR, but given the fact that NHR does not perfectly map on to government classifications of marginalization but instead reflect psychological tendencies associated with potential marginalization, as NHR increases, the probability of marginalization, such as the probability of dropping out of education or the workforce, also increases.

Future analyses should examine whether the psychological tendencies we measured with the NHR scale are associated with the risk of being marginalized in other postindustrial societies besides Japan, such as the United States, the UK, and South Korea. For now, we assume the NHR factors are applicable to other post-industrial societies, because in such societies young people are increasingly facing uncertainties about what they can do (Arnett, 2004). Having said that, the Freeter lifestyle preference (Factor 1) might be more specific to the idea of cultural marginalization in Japan since it is related to a preference to not conform to interdependent and hierarchical social norms, but the other two factors (low self-competence and a lack of clear goals) should be relevant to psychological tendencies associated with marginalization in other post-industrial societies. Thus, future research should test the cross-cultural validity of this scale.

Finally, from a cultural psychological point of view, we assume that even if the risk factors might be quite similar across cultures, the behavioral consequences of this risk may be different, since each cultural context affords different behavioral patterns. In Japan, marginalized psychological tendencies are more likely to be associated with withdrawal and “internalizing” tendencies, perhaps because it is normative to suppress strong emotions (Murata et al., 2013) and to not disturb others too much (Norasakkunkit et al., 2012). In other societies, marginalized youth may exhibit more “externalizing” tendencies such as joining a gang, drug abuse, showing emotional outbursts, or even engaging in school shootings (see Merton, 1938 discussion of those who retreat from society). This means that behavioral tendencies that are associated with marginalization may also be culturally shaped or culturally scripted. Thus far, cultural psychological research has focused on identifying cross-cultural differences in culturally normative behavior (i.e., interdependence in Japan and independence in the US), but it may now be time to consider how culture may shape the behavioral tendencies of those living on the fringes of society as well.

### Conflict of interest statement

The Guest Associate Editor Tuukka Hannu Ilmari Toivonen declares that, despite having collaborated on the Research Topic (Cultural change: Adapting to it, coping with it, resisting it, and driving it.) with authors Yukiko Uchida and Vinai Norasakkunkit, the review process was handled objectively and no conflict of interest exists.

## Footnotes

^1^Freeter ia a combination of the word “Freelance” and the German word for part time worker or “Arbeiter.”

^2^Before controlling NHR score, a simple ANOVA analysis found that SWB was higher in the normal sample than the categorical NEET and the categorical Hikikomori. The main effect was significant *F*_(2, 7722)_ = 81.60, *p* < 0.0001, η*p*2 = 0.02 and the differences between three categories were also significant across each other. Thus, SWB was higher in normal people (5.80) > than NEETs (4.40) > the Hikikomori (3.43). But in the regression analysis we reported, it was suggested that the power of the categorical index to explain SWB differences disappeared when we controlled the NHR.
