# Supplementary material for: The NEET and Hikikomori spectrum: Assessing the risks and consequences of becoming culturally marginalized
Source: Front Psychol. 2015 Aug 18;6:1117. doi: 10.3389/fpsyg.2015.01117 (PMC4540084; doi:10.3389/fpsyg.2015.01117)
Supplement: Supplementary file 1 [file DataSheet1.DOCX]

Appendix 1. Books used for sampling of items

| Title | Author | Year of publication |
| --- | --- | --- |
| Young people and labor market precarity: Freeter, NEET, unemployed in Japan and the UK | Inui, A. et al. | 2006  Otsuki Shoten |
| Sociology of Freeters and NEETs | Taromaru, H. (Ed). | 2006 Sekaisisosha |
| NEET | Genda, Y., & Maganuma, M. | 2004, Gentosha |
| Don’t say “NEET” | Honda, Y., Naito, A., & Goto, K. | 2006  Kobunsha |
| Young people trapped in freeter status | Hori, Y. | 2007, Keiso shobo |
| If your child becomes NEET | Genda, Y., Kosugi, R., &The Japan Institute for Labor Policy and Training | 2005, NHK publishing |
| Hikikomori and family trauma | Hattori, Y. | 2005，NHK Publishing |
| Social withdrawal: a never ending adolescence | Saito, T. | 1998，PHP research Institute |
| Those who believe that they have lost: a social theory of NEET and socially withdrawn | Saito, T. | 2005, chuo Koron shinsha |
